# Tuberculosis among older adults in Zambia: burden and characteristics among a neglected group

**DOI:** 10.1186/s12889-017-4836-0

**Published:** 2017-10-12

**Authors:** Jenna Coffman, Pascalina Chanda-Kapata, Ben J. Marais, Nathan Kapata, Alimuddin Zumla, Joel Negin

**Affiliations:** 1 School of Public Health, University of Sydney, Sydney, Australia; 2 Department of Disease Surveillance, Control and Research, Ministry of Health, Lusaka, Zambia; 3 Marie Bashir Institute for Infectious Diseases and Biosecurity, University of Sydney, Sydney, Australia; 4 Children’s Hospital at Westmead, University of Sydney, Sydney, Australia; 5 National TB and Leprosy Control Program, Lusaka, Zambia; 6 Division of Infection and Immunity, Department of Infection, University College London, London, UK

**Keywords:** Tuberculosis, Older adults, Zambia

## Abstract

**Background:**

The 2010 Global Burden of Disease estimates show that 57% of all TB deaths globally occurred among adults older than 50 years of age. Few studies document the TB burden among older adults in Southern Africa. We focused on adults older than 55 years to assess the relative TB burden and associated demographic factors.

**Methods:**

A cross sectional nationally representative TB prevalence survey conducted of Zambian residents aged 15 years and above from 66 clusters across all the 10 provinces of Zambia. Evaluation included testing for TB as well as an in-depth questionnaire. We compared survey data for those aged 55 and older to those aged 15–54 years. Survey results were also compared with 2013 routinely collected programmatic notification data to generate future hypotheses regarding active and passive case finding.

**Results:**

Among older adults with TB, 30/ 54 (55.6%) were male, 3/27 (11.1%) were HIV infected and 35/54 (64.8%) lived in rural areas. TB prevalence was higher in those aged ≥55 (0.7%) than in the 15–54 age group (0.5%). Males had higher rates of TB across both age groups with 0.7% (15–54) and 1.0% (≥55) compared with females 0.4% (15–54) and 0.6% (≥55). In rural areas, the prevalence of TB was significantly higher among older than younger adults (0.7% vs 0.3%), while the HIV infection rate was among TB patients was lower (11.1% vs 30.8%). The prevalence survey detected TB in 54/7484 (0.7%) of older adults compared to 3619/723,000 (0.5%) reported in 2013 programmatic data.

**Conclusion:**

High TB rates among older adults in TB endemic areas justify consideration of active TB case finding and prevention strategies.

## Background

Global efforts to reduce mortality from tuberculosis (TB) has shown solid progress with mortality rates decreasing over the past decade [1]. Programs on prevention, screening and treatment have contributed to these declines among certain groups such as those vulnerable to developing TB such as children [2], those living with HIV [3], and people with diabetes [4]. The World Health Organization estimates that the global case detection rate reached 64% in 2013 and that treatment success rates were above 85% [1]. However, despite the progress achieved and increased attention on certain groups, there has been ongoing neglect of older adults – those aged 50 years and older, especially in low and middle-income countries [5].

Older adults’ vulnerability to TB has long been recognised [6]. Data from the 2015 Global Burden of Disease studies show that 62% of all tuberculosis deaths globally occurred among adults older than 50 [7, 8]. The World Health Organization acknowledges that some regions have seen a progressive increase in the prevalence of TB with age [1] which has been confirmed in studies from countries such as China [9, 10], India [11, 12], and the Americas [13, 14]. But in Southern Africa, which remains the region of the world most affected by tuberculosis with the highest incidence and prevalence of the disease [8], the age distribution of the disease is often poorly defined. The TB challenge in Southern Africa is exacerbated by the HIV epidemic – which itself is ageing with the success of anti-retroviral treatment [15, 16].

Few studies exist on the TB burden among older adults in Southern Africa. Karstaedt and Bolhaar found a high burden of TB among the elderly in Soweto and that 50% of the older adults with TB had extra-pulmonary tuberculosis [17]. Blaser and colleagues’ age-structured transmission model for Cape Town found that simulated TB notification rates gradually increased from the age of 30 years [18].

Understanding the burden of TB among older adults is important due to specific features of the disease and its diagnosis among this age group. Older adults are more vulnerable to developing TB disease and are more likely to develop atypical forms of TB such as tuberculous meningitis, and sputum smear-negative pulmonary involvement which are often harder to diagnose and treat [19–21]. Diagnosis is also more challenging as common symptoms used for diagnosis are less prominent in older populations [21] and the common Mantoux skin test tends to produce false negative results in older patients. [21–24] Furthermore, vulnerability among older adults is increased due to increased rates of compromised immune systems due to co-morbidity such as diabetes [25] and cancers [5].

We therefore analysed the 2013–14 Zambia TB prevalence survey data focusing specifically on older adults aged 55 years and above, in order to better understand TB burden and associated demographic factors in this population in a high-burden country. In addition, we sought to compare the survey data to routine national notification data. Zambian TB incidence has been estimated as of 2015 at 205.9 per 100,000 population with 3573 deaths (22.0 per 100,000 population)by the Global Burden of Disease study [8].

## Methods

The 2013–14 Zambia TB prevalence survey was a cross sectional survey of the prevalence of TB among residents aged 15 years and above from 66 clusters across all 10 provinces of Zambia. In the initial census, 98,458 participants were identified. Symptom and chest x-ray screening was offered to all survey participants after obtaining consent. There was a 84.1% participation rate from the participants originally identified. Those who reported any relevant symptoms and/or were found to have an abnormal chest x-ray underwent an in-depth questionnaire. In addition, they were requested to submit a spot and morning sputum sample for MTB identification which was identified by either liquid culture (using MGIT BACTEC 960™). Indeterminate sputum smear samples where there was no conclusive culture results were subjected to Xpert MTB Rif. HIV counselling and testing was offered to all survey participants. The detailed national TB prevalence survey procedures and HIV testing procedures are reported elsewhere [26, 27].

In this analysis, survey data for those aged 55 and older was extracted from the main survey database. The variables extracted included participant age, residence, employment status, education level and socio-economic status. The prevalence of TB and socio demographic characteristics among those aged 55 years and above were compared with those aged 15–54 years. Odds ratios (OR) and confidence intervals were produced using logistic regression, accounting for clustered sampling with 66 primary sampling units (PSU). Socioeconomic status was determined by deriving wealth indices using data collected on household assets, housing characteristics and access to utilities. This was achieved using the standard asset ownership questionnaire used in the Zambia Demographic Health Surveys,

The survey data were then compared with 2013 routinely collected data to ascertain whether there was a difference in rates of TB obtained between the two forms of data collection. An implied prevalence based on total population disaggregated by age and province was calculated. The 2013 routinely collected data was available from Ministry of Health national TB notification reports.

Data were analysed with SPSS version 22 software (IBM Corp. Released 2013. IBM SPSS Statistics for Mac, Version 22. AMonk, NY: IBM Corp.) and Stata Statistical Software: Release 12 (StataCorp, 2011). Overall TB prevalence rates were calculated; additional subgroup analyses were done to calculate specific prevalence rates by age, sex, geographical location, employment status, education level, and HIV status. Health seeking behaviour was analysed to assess access to health facilities.

The Zambian National TB prevalence survey study protocol was cleared by the University of Zambia Biomedical Research Ethics Committee (UNZABREC) No: 020–08-12.

## Results

Data for the Zambia national tuberculosis prevalence survey was collected between August 2013 and July 2014. The study identified 98,458 individuals in 66 clusters. Of these, 54,830 (55.7%) were residents and were aged 15 years and older and were therefore eligible to participate. Of these, 46,099 (84.1%) underwent symptom screening, while 45,633 (99%) were screened by chest x-ray (CXR) at field level. From these, 4453 had positive symptom screenings while 3758 (8.2%) had an abnormal CXR. The mean age of the participants was 36.1. Of all respondents, 16.2% were aged 55 and older.

Among participants, the overall prevalence of TB was higher in those aged 55 and older (0.7%) than in the 15–54 age group (0.5%) (Table 1). Males had significantly higher rates of TB across both age groups with 0.7% (15–54) and 1.0% (55+) compared with females (0.4% (15–54) and 0.5% (55+)) (Table 1). In rural areas, the prevalence of TB was significantly higher among older adults than younger adults. Prevalence was highest in the Copperbelt province for both younger and older adults. The unemployed had a higher prevalence of TB across both age groups with 0.6% (15–54) and 0.8% (55+) compared with the employed (0.5% (15–54) and 0.6% (55+)). Tertiary educated older adults had the highest prevalence of TB (1.4%) across both age groups. TB prevalence across socioeconomic quintiles did show a trend with the highest rates of TB seen in the 3rd and 4th quintiles across both groups with slightly higher prevalence among older adults.

**Table 1:** Number of people screened for tuberculosis in the Zambian national tuberculosis prevalence survey 2013–14, proportion of people diagnosed with tuberculosis and association of age group (15–54 years and 55 years and above) with demographic characteristics

Demographic characteristic		15–54		55+		Odds Ratios (95% Confidence intervals)
		Population	Number of Cases	Population	Number of Cases	
Overall		38,619	211 (0.5%)	7484	54 (0.7%)	1.32 (0.95–1.86)
Sex	Male	16,315	122 (0.7%)	3145	30 (1.0%)	1.28 (0.80–2.04)
	Female	22,304	89 (0.4%)	4339	24 (0.6%)	1.39 (0.89–2.17)
Location	Urban	13,783	126 (0.9%)	2274	19 (0.8%)	0.92 (0.58–1.44)
	Rural	24,836	85 (0.3%)	5210	35 (0.7%)	1.97 (1.35–2.89)
Province	Central	2403	12 (0.5%)	503	1 (0.2%)	0.40 (0.08–1.97)
	Copperbelt	6608	74 (0.1%)	1628	19 (1.2%)	1.04 (0.51–2.14)
	Eastern	5990	8 (0.1%)	1167	4 (0.3%)	2.57 (1.86–3.55)
	Luapula	3013	7 (0.2%)	646	2 (0.3%)	1.33 (0.31–5.69)
	Lusaka	7414	63 (0.9%)	1128	8 (0.7%)	0.83 (0.49–1.42)
	Muchinga	1052	2 (0.2%)	199	0 (0.0%)	NA
	Northern	3296	17 (0.5%)	594	10 (1.7%)	3.31 (1.81–6.05)
	North Western	2310	9 (0.4%)	530	4 (0.8%)	1.94 (1.24–3.06)
	Southern	5089	11 (0.2%)	594	4 (0.7%)	3.12 (0.74–13.19)
	Western	1444	8 (0.6%)	495	2 (0.4%)	0.73 (0.29–1.82)
Employment Status ^a^	Unemployed	17,951	102 (0.6%)	2779	22 (0.8%)	1.40 (0.84–2.33)
	Employed	20,405	109 (0.5%)	4494	29 (0.6%)	1.21 (0.82–1.79)
Highest Level of Education	No Schooling	2172	8 (0.4%)	2107	18 (0.9%)	2.33 (0.99–5.49)
	Primary	16,759	98 (0.6%)	3916	24 (0.6%)	1.05 (0.61–1.79)
	Secondary	18,081	100 (0.6%)	1243	9 (0.7%)	1.32 (0.68–2.55)
	Tertiary	1576	5 (0.3%)	218	3 (1.4%)	4.35 (0.73–25.85)
Socioeconomic Status ^b^	Lowest	7374	31 (0.4%)	1474	4 (0.3%)	0.65 (0.26–1.63)
	Lower	6765	29 (0.4%)	1366	8 (0.6%)	1.37 (0.69–2.73)
	Middle	7099	51 (0.7%)	1370	14 (1.0%)	1.43 (0.64–3.18)
	Higher	5914	39 (0.7%)	1123	14 (1.2%)	1.90 (0.93–3.91)
	Highest	4991	25 (0.5%)	849	3 (0.4%)	0.70 (0.21–2.40)

Denominator is the total number of eligible individuals who took part in the study and fit into the relevant category being assessed

^a^Employed includes occasional seasonal workers, government employees, private sector employees, self-employed, working on own land. Unemployed includes unemployed, students and housewife/homemakers

^b^Socioeconomic status was determined by deriving wealth indices using data collected on household assets, housing characteristics and access to utilities. This was achieved using the standard asset ownership questionnaire used in the Zambia Demographic Health Surveys

Of older adults diagnosed with TB the mean age was 66 (Table 2). Of those older adults living with TB, 55.6% were male. Compared to those aged 15–54, older adults living with TB were much more likely to be living in rural areas. Of older adults that were TB positive, 11.1% were also HIV positive.

**Table 2:** Characteristics of tuberculosis positive people identified in the Zambian national tuberculosis prevalence survey, 2013–14, by age group (15–54 years and 55 years and above)

Characteristics		Younger (15–54 yrs) *n* = 211	Older (≥55 yrs) *n* = 54
Mean age		36	66
Sex	Male	122 (57.8%)	30 (55.6%)
Location	Urban	126 (59.7%)	22 (40.3%)
	Rural	74 (35.2%)	35 (64.8%)
Education	No schooling	8 (3.8%)	18 (33.3%)
	Primary	98 (46.5%)	24 (44.4%)
	Secondary	100 (47.4%)	9 (16.7%)
	Tertiary	5 (2.4%)	3 (5.6%)
Socioeconomic status		*n* = 175	*n* = 43
	Lowest	31 (17.7%)	4 (9.3%)
	Lower	29 (16.6%)	8 (18.6%)
	Middle	51 (29.1%)	14 (32.6%)
	Higher	39 (22.3%)	14 (32.6%)
	Highest	25 (14.3%)	3 (7%)
Number in Household		*n* = 184	*n* = 53
	1–4	45 (24.5%)	13 (24.5%)
	5–8	101 (54.9%)	28 (52.8%)
	9 + −12	38 (20.7%)	12 (22.7%)
Family member treated for TB		*n* = 210	*n* = 51
	Yes	38 (18.1%)	3 (5.9%)
HIV-infected (among those with TB)		(*n* = 107)	(*n* = 27)
		33 (30.8%)	3 (11.1%)
Transport used to visit nearest facility		n = 211	n = 54
	Walk	188 (89.1%)	42 (77.8%)
	Bicycle	8 (3.8%)	6 (11.1%)
	Other	15 (7.1%)	0 (0%)
Travel time to nearest facility	< 1 h	173 (82%)	38 (70.7%)
	1 to 3 h	34 (16.1%)	15 (27.8%)
	>3 h	4 (1.9%)	1 (1.9%)

We compared the survey data with 2013 routinely collected data to ascertain whether there was a difference between the outcomes from the two forms of data collection (Table 3). Overall rate of TB among those aged 15–54 was the same between the survey and routine collection but for older adults (55+), survey prevalence was higher (0.7%) compared to the implied prevalence from routine collection (0.5%). This was particularly pronounced for older males where survey prevalence was 1.0% compared to 0.6% from routine data. Data from Copperbelt Province found double the TB prevalence among older adults in the survey than found in routine data collection and Northern Province – the largest and most remote province – found a much higher rate of TB through the survey as through routine data.

**Table 3:** Tuberculosis prevalence among older adults (aged 55+) based on survey results compared to routinely collected programmatic data

			Survey	Routine data collection
Overall prevalence		15–54	0.5% (38,816)	0.5% (6,263,000)
		55+	0.7% (7512)	0.5% (743,000)
Sex	Male	15–54	0.7% (16,415)	0.6% (3,148,000)
		55+	1.0% (3156)	0.6% (349,000)
	Female	15–54	0.4% (22,401)	0.4% (3,128,000)
		55+	0.6% (4356)	0.4% (393,000)
Provinces	Central	15–54	0.5% (2471)	0.3% (618,688)
		55+	0.2% (51)	0.3% (70,490)
	Copperbelt	15–54	1.1% (6608)	0.7% (955,808)
		55+	1.2% (1628)	0.6% (108,899)
	Eastern	15–54	0.1% (5990)	0.2% (833,373)
		55+	0.3% (1167)	0.3% (94,950)
	Luapula	15–54	0.2% (3081)	0.3% (467,980)
		55+	0.3% (660)	0.5% (53,319)
	Lusaka	15–54	0.9% (7414)	1.1% (1,073,110)
		55+	0.7% (1128)	0.9% (122,264)
	Northern	15–54	0.5% (3302)	0.1% (858,685)
		55+	1.7% (594)	0.2% (97,834)
	North Western	15–54	0.4% (2310)	0.3% (344,753)
		55+	0.8% (530)	0.3% (39,279)
	Southern	15–54	0.2% (5107)	0.3% (784,115)
		55+	0.7% (597)	0.5% (89,338)
	Western	15–54	0.6% (1444)	0.5% (430,184)
		55+	0.4% (495)	1.0% (49,013)

Population figures from 2010 Zambian census

## Discussion

Analysis of the Zambia TB prevalence survey found high rates of TB among those aged 55 years and older compared to younger age groups. This difference was consistent among men and women and was particularly pronounced among older adults living in rural areas. Older adults in higher socioeconomic status bands had higher TB prevalence than younger adults in those bands. HIV prevalence was lower among older adults living with TB than among younger adults living with TB suggesting that co-morbidity is less of a driver of TB among the older population. The rates of TB were found to be higher among older adults in the survey than what was found from notification data that was collected through routine services, suggesting that some older adults are being missed by currently available health services.

The fact that the high burden of TB among older adults is not as associated with HIV infection is notable. In 2012, a national tuberculosis survey done in Tanzania demonstrated an epidemic shift from young HIV-infected to older HIV-uninfected people [28]. The highest TB prevalence rate found in that survey was among those aged 45 years and older in whom HIV prevalence rates were generally lower. Though HIV prevalence among older adults is increasing [16, 29], there is need to better understand the determinants and drivers of the TB burden among the older population.

The suggested burden of undiagnosed TB among older adults has been found in other LMIC settings as well. In urban Cambodia, a community-based active case finding project found that 26% of new cases found were older than 65 which was double the proportion found through passive case finding [30, 31]. Together, this suggests that the current emphasis on HIV and TB co-morbidity is not sufficient to address the challenges of this under-diagnosed pool of older HIV-uninfected older adults who may have particular challenges with regard to health access due to poverty, rural living, mobility difficulties [32] and ageist stigma [33]. There is currently limited evidence regarding successful interventions for the improvement of diagnosis of TB among older adults in LMICs [34].

Relatively high rates of TB among older adults challenge the current TB response and require new data and thinking. Li and colleagues’ recent scoping review [34] identified a number of causal pathways to improve TB prevention and services among older people. While the review focused on articles from high-income countries [34], some of the interventions might be relevant in low- and middle-income settings. Screening of latent TB infection for example reduced the risk of reactivation [35, 36] and the review authors promoted active systematic screening for high-risk groups to reduce the delay in diagnosis and containment. Improving the health of older adults living with TB is important not only for their health but, additionally, older carers with TB pose a risk to the young children in their care. Therefore, programs have the potential to be framed as a lifespan intervention.

### Limitations

We acknowledge the limitations of the study and subsequent analysis. Diagnosis of TB among older adults is difficult [21]. Further, due to the cross-sectional study design, we were unable to separate the independent influences of age, socioeconomic status, geographical location, education level, and employment status on TB prevalence in this study. Some of the differences we found with regard to younger versus older adults may be attributable to such confounders; we were unable to include a regression analysis to further analyse the relationship of age, TB status and associated risk factors due to the small numbers of those with TB for the over 55 age group. Further analyses will do so now that this preliminary analysis has generated hypotheses. Additionally, we acknowledge that the category of 15–54 is a large age group and there are likely substantial differences within that group – but given our focus on older adults specifically, we have not performed analyses within that younger age range.

We acknowledge that comparing the study data with the routinely collected data is not comparing the same measurements but have included for interest and to encourage further research. Additionally, we acknowledge that 16.2% of respondents being 55+ is an over–representation of this age group compared with the Zambian population but this also means that the data for this group was more robust than it would have been otherwise.

## Conclusion

The findings from this national survey suggest that the national tuberculosis program in Zambia and similar settings should prioritise active case finding among older adults. In addition, prevention activities should be specifically targeted to older adults where they live and socialise [37].
